# Formation of precipitates in off-stoichiometric Ni–Mn–Sn Heusler alloys probed through the induced Sn-moment

**DOI:** 10.1039/d3ra01420g

**Published:** 2023-06-15

**Authors:** Benedikt Eggert, Aslı Çakır, Damian Günzing, Nicolas Josten, Franziska Scheibel, Richard A. Brand, Michael Farle, Mehmet Acet, Heiko Wende, Katharina Ollefs

**Affiliations:** a Faculty of Physics, Center for Nanointegration Duisburg-Essen (CENIDE), University of Duisburg-Essen Lotharstr. 1 47057 Duisburg Germany Benedikt.Eggert@uni-due.de; b Department of Metallurgical and Materials Engineering, Muğla Sıtkı Koçman University 48000 Mugla Turkey; c Functional Materials, Institute of Materials Science, Technische Universität Darmstadt 64287 Darmstadt Germany

## Abstract

The shell-ferromagnetic effect originates from the segregation process in off-stoichiometric Ni–Mn-based Heusler alloys. In this work, we investigate the precipitation process of L2_1_-ordered Ni_2_MnSn and L1_0_-ordered NiMn in off-stoichiometric Ni_50_Mn_45_Sn_5_ during temper annealing, by X-ray diffraction (XRD) and ^119^Sn Mössbauer spectroscopy. While XRD probes long-range ordering of the lattice structure, Mössbauer spectroscopy probes nearest–neighbour interactions, reflected in the induced Sn magnetic moment. As shown in this work, the induced magnetic Sn moment can be used as a detector for microscopic structural changes and is, therefore, a powerful tool for investigating the formation of nano-precipitates. Similar research can be performed in the future, for example, on different pinning type magnets like Sm-Co or Nd-Fe-B.

## Abstract 2

The shell-ferromagnetic effect originates from the segregation process in off-stoichiometric Ni–Mn-based Heusler.

## Graphical abstract

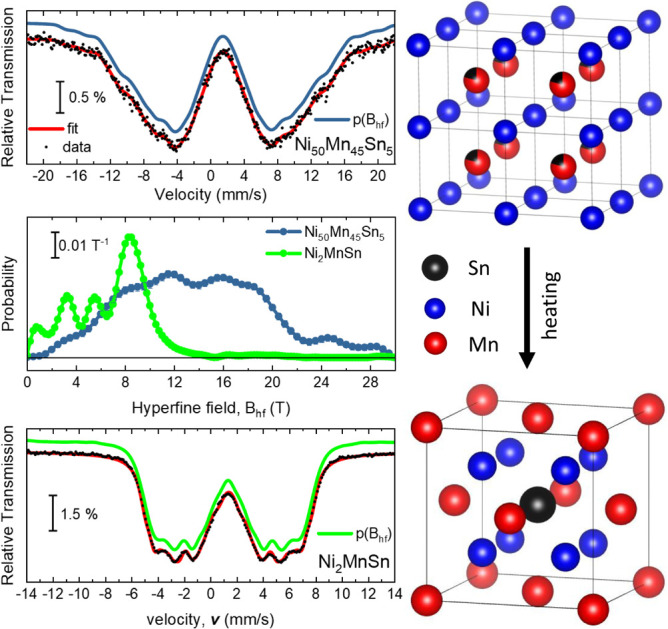

## 1 Introduction

Due to the multifaceted phase diagram^1,2^ of magnetic Heusler alloys, this material class possesses a variety of interesting phenomena.^3^ For example, NiMn–Heusler alloys show a magnetostructural phase transition^4^ or intrinsic exchange bias^5^ due to the presence of mixed magnetic interactions (antiferromagnetic and ferromagnetic).^6–10^ Besides, it shows the possibility of adjusting the magnetic anisotropy energy, *e.g.* by interstitial doping^11^ and, therefore, serves as a prototype system for investigations of fundamental physical phenomena such as structural disorder.^12^ These properties make Heusler alloys interesting for serveral applications, for example, in the area of magnetic shape-memory,^13^ magnetocalorics^14^ and spintronics.^15^ Heusler alloys have a huge potential for applications, however, off-stoichiometric variations of Heusler alloys suffer due to a tendency of segregation. Sokolovskiy *et al.*^10^ recently performed DFT-calculations and showed that off-stoichiometric Mn-rich Ni_2_Mn_1+*x*_(In,Sn,Al)_1−*x*_ compounds are unstable at low temperatures and decompose into a dual phase system. However, it is possible to utilize this process, as it will be discussed in the following. The shell-ferromagnetic effect is a newly achieved property of the off-stoichiometric Heusler compound, which is less well studied and occurs in Mn-rich antiferromagnetic (AF) Heusler-based compounds^16^ and opens paths to different functionalities. This effect occurs when Ni_50_Mn_45_Z_5_ (Z: Al, Ga, In, Sn, Sb) decomposes into cubic L2_1_ ferromagnetic (FM) Heusler Ni_50_Mn_25_Z_25_ and L1_0_-ordered AF Ni_50_Mn_50_ during temper-annealing at temperatures around 600 K < *T*_A_ < 750 K, where *T*_A_ is the annealing temperature. By applying a magnetic field during the annealing process, nano-precipitates are formed within a strongly pinning AF matrix, originating from Ni–Mn and off-stoichiometric Ni_50_Mn_45_Z_5_. A collection of these nano precipitates in a macroscopic sample gives rise to a partially compensated magnetic response to an applied magnetic field, which has been demonstrated in a video.^17^ The observed pinning mechanism implies that the formed precipitates could form building blocks for high-performance and lightweight permanent magnets of unsurpassed coercivity. The magnetic moment at the interface of Ni–Mn and Ni_2_MnSn, becomes pinned in the direction of the applied magnetic field during annealing so that the field-dependence up to 9 T appears as a vertically shifted hysteresis loop, while it is a minor loop within a major loop with a coercive field exceeding 5 T.^18^ The remanent magnetisation of the loop is always positive and only reorientates entirely in fields exceeding 20 T (*T* < 550 K). The core structure of the precipitate (Ni_2_MnZ) is, however, magnetically soft, and the spins rotate freely in the direction of an applied magnetic field. These structures were first found as a result of decomposing Ni_50_Mn_45_In_5_ (ref. 19) or Ni_50_Mn_45_Ga_5_ (ref. 16) at 650 K in a magnetic field. For the effective compensation of the magnetisation, the surface-to-volume ratio of the precipitate is important. For the case of a large surface-to-volume ratio, the magnetisation of the Ni_2_MnZ-nano-precipitates can be compensated by the Ni–Mn surrounding. For larger Ni_2_MnZ-precipitates, the Ni–Mn-surrounding is not sufficient for the compensation of Ni_2_MnZ spins, and the shell-ferromagnetic effect does not occur. Scherrer analysis indicates a precipitate size of 3–5 nm for Ni_50_Mn_45_In_5_ annealed at 650 K, corresponding to a surface-to-volume ratio^20^ of 1.2–2 nm^−1^, while for Ni_50_Mn_45_Sb_5_ the precipitate size is in a similar range of 5–10 nm.^21^ Besides the potential use case in permanent magnets, these magnetically pinned precipitates can be used in materials possessing a first-order magnetostructural phase transition,^22^ where the precipitates may serve as a nucleation site for the phase transition. In this case, the precipitates may induce a local strain field that can energetically favour the martensite–austenite transition. This mechanism has the potential to improve magnetocaloric properties^23,24^ of this compound or can increase the mechanical stability.^25–30^

Within the current work, we report on our recent findings characterising Ni–Mn–Sn precipitates and show that ^119^Sn-Mössbauer spectroscopy is an ideal technique to study the precipitate formation due to the possibility to probe nearest–neighbour interactions through the Sn nuclei. Therefore, we can observe phases with a short-range ordering otherwise absent or difficult to detect with XRD. We will show this trend by comparing our spectroscopic insights with X-ray diffraction results that resolve long-ranged ordering. ^119^Sn-Mössbauer spectroscopy tracks the formation of stoichiometric Ni_2_MnSn clusters inside an antiferromagnetic Ni_50_Mn_45_Sn_5_ matrix for mild annealing temperatures. Here, we indirectly probe the induced Sn-moment and use this spectroscopic feature as a detector for the structural transition, without the need for another tracing dopant used for example in ref. 31, which leads to local distortions and effects the physical properties. On the other hand, X-ray diffraction is a well-known and effective tool to probe the long-range ordering of the whole sample volume.

## 2 Results & discussion

**Fig. 1:**
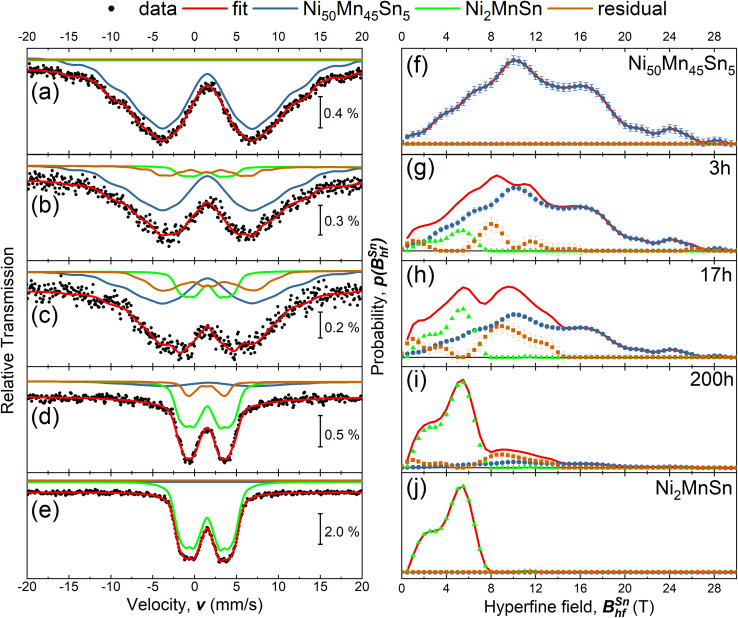
(a)–(e) Zero field Sn-Mössbauer spectroscopy measurements of the as-prepared Ni_50_Mn_45_Sn_5_, Ni_50_Mn_45_Sn_5_ heated at 650 K for *t*_A_ = 3 h, 17 h, 200 h and for comparison an as-prepared stoichiometric Ni_2_MnSn Heusler. All measurements were performed at room temperature. The individual spectra of the annealed states can be described by a linear combination of the spectra from the precursor materials (Ni_50_Mn_45_Sn_5_ & Ni_2_MnSn) an additional residual distribution of hyperfine fields *p*(*B*^Sn^_hf_). Additional details concerning the analysis of the Mössbauer spectra are discussed in the text. (f)–(j) Combined hyperfine field distribution *p*(*B*^Sn^_hf_) for the respective measurements (red line), obtained by a combination of the sub spectra (blue, green and brown dots).

In order to test the sensitivity of the Sn hyperfine field to structural changes, we performed ^119^Sn-Mössbauer spectroscopy at room temperature on the AFM-ordered off stoichiometric L1_0_-alloy Ni_50_Mn_45_Sn_5_ and stoichiometric FM-ordered L2_1_ Ni_2_MnSn alloy (see Fig. 1(a) & (e)).^32^ The magnetic ordering of the samples leads to the lifting of the degeneracy of the ^119^Sn hyperfine levels and the occurrence of a sextet structure. In the following both spectra are described by a hyperfine field distribution *p*(*B*^Sn^_hf_) which is (shown in Fig. 1(f) & (j)) for the L1_0_ and L2_1_-ordered alloy, respectively. The spectrum of the L1_0_-ordered alloy (see Fig. 1(a)) possesses a relatively broad hyperfine field distribution ranging from 0 T to almost 28 T (see Fig. 1(f)) with an average hyperfine field 〈*B*^Sn^_hf_〉 of 12.1 T. The broad distribution of hyperfine fields indicates different local surroundings around the ^119^Sn-nuclei. In contrast, the L2_1_-ordered alloy feature a smaller distribution with distinct fine structure at 2 and 6 T leading to an average hyperfine field 〈*B*^Sn^_hf_〉 of 3.9 T. The present distribution of hyperfine fields indicates the presence of structural or magnetic disorder and expresses small variations from the L2_1_-crystal structure, since for the L2_1_ structure, we would assume the presence of a single Sn-environment leading also to a single spectral contribution. This phenomenon has been extensively discussed in a recent work on the effect of magnetic and anti-site disorder for the Sn-partial phonon density of states in Ni_2_MnSn,^33^ and goes beyond the scope of the current work. These defects could be present in the form of anti-site disorder between Mn and Sn atoms caused due to the slight deviation of the perfect 2–1–1 stoichiometry (see Table 2). By comparing these two compositions, we can conclude that replacing Mn with Sn dilutes the absolute magnetic moment and, therefore, reduces the ^119^Sn hyperfine field. Accordingly, the major contribution in the hyperfine field distribution (see Fig. 1(f) & (j)) shifts towards smaller fields and reflects the decreased Sn moment. Here, we cannot determine the exact change of the Sn magnetic moment due to the complex relationship between the magnetic moment and the hyperfine field. For Sn, the proportionality constant *A* between the Sn moment *μ*_Sn_ and the Sn hyperfine field *B*^Sn^_hf_ depends on different materials properties, *e.g.* the anisotropy of the system.^34–36^ Furthermore, the effective hyperfine field measured at the ^119^Sn nucleus is composed of several terms. There are direct (dipolar) and indirect (transfered hyperfine field from neighboring atom to ^119^Sn nucleus) terms. There is also the possibility that the Sn atom is itself polarized from its surroundings and generates a direct (contact) hyperfine field. Without further information (for example XMCD spectra of Sn), we can only conclude that the measured *B*^Sn^_hf_ is the sum of relevant contributions. Therefore, the determination of the magnetic Sn-moment *μ*_Sn_ is beyond the scope of this work.

In the following, the decomposition process will be investigated. As stated in previous investigations,^16,19,20^ the decomposition process in the off-stoichiometric Heusler compound follows the route1
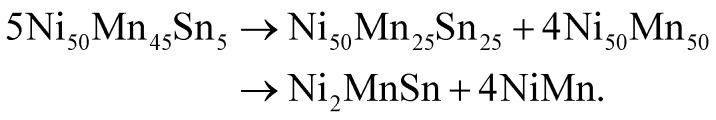


In the following, we assume that for finite annealing times, the overall decomposition process can be described with an additional residual component, leading to a modification of eqn (1) to2
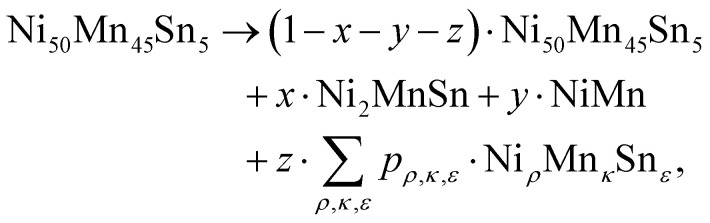
where Ni_*ρ*_Mn_*κ*_Sn_*ε*_ is the residual Sn-containing phase with unknown stoichiometry, while *x*, *y*, *z*, and *p*_*ρ*,*κ*,*ε*_ is the respective molar fraction of the respective phase.

Due to excitation of the nuclear resonance, ^119^Sn-Mössbauer spectroscopy probes only Sn-containing phases. Therefore, in the discussed case, one can track the temporal evolution of the decomposition process by identifying spectral fingerprints for the respective Sn-containing phases. Based on eqn (2), it is possible to probe the initial compound Ni_50_Mn_45_Sn_5_, the Ni_2_MnSn-core structure, and the Sn-containing residual phase Ni_*ρ*_Mn_*κ*_Sn_*ε*_, while the formation of Ni–Mn can not be observed *via*^119^Sn-Mössbauer spectroscopy, due to the missing Sn-content. With the spectral fingerprint of the initial and the core-precipitate compound (see Fig. 1(a) & (e)) the experimental spectra of the decomposed state can be described by a least-squares fitting routine, assuming a superposition of the known theoretical models (for Ni_50_Mn_45_Sn_5_ and Ni_2_MnSn), while an additional hyperfine field distribution *p*(*B*^Sn^_hf_) describes the residual spectral contributions arising from unknown compositions. Based on this model, we can model the ^119^Sn Mössbauer spectra for annealing times *t*_A_ of 3 h, 17 h, and 200 h (see Fig. 1(b)–(e)), while Fig. 1(g)–(i) depicts the obtained hyperfine field distributions and the overall sum. Here the annealing temperature *T*_A_ was chosen to be 650 K, since at these temperatures similar studies on Ni_50_Mn_45_In_5_ (ref. 20) or Ni_50_Mn_45_Sb_5_ (ref. 21) indicate that the size of the precipitate is almost temperature independent and below 10 nm – resulting in a sizeable surface-to-volume ratio. The size of the precipitates leads to the intrinsic compensation of the respective magnetic moments in the Ni–Mn shell and Ni_2_MnSn core – resulting in the shell-ferromagnetic effect^19^ and the occurrence of these large coercivity fields.^18^ The comparison of the hyperfine field distributions reveals the shift of the maximum hyperfine field towards smaller values with increasing annealing times *t*_A_, while after annealing for 200 h, the majority of the hyperfine field contribution originates from stoichiometric Ni_2_MnSn. The relative spectral area (see Table 1) supports this trend: with increasing annealing duration, the Ni_2_MnSn and residual contribution increase. Here, we want to mention two further aspects. Our room temperature measurements do not indicate a significant contribution of superparamagnetic Ni_2_MnSn-clusters, since we only observe a minor contributions at low hyperfine fields in the residual phase. On the other hand, measurements performed above the Curie temperature of Ni_2_MnSn (not shown here) show similar spectral contributions to the previously discussed room temperature measurements, but show a major singlet contribution, indicating the collapse of long-range magnetic ordering.

**Table 1:** Relative spectral area obtained from the hyperfine field distribution *p*(*B*_hf_) for the different components

*t*_A_ (h)	Ni_50_Mn_45_Sn_5_ (%)	Ni_2_MnSn (%)	Residual (%)
0	100	0	0
3	80.8(1.8)	11.3(1.1)	7.9(9)
17	57.9(2.3)	19.8(2.1)	22.3(1.6)
200	15.2(1.5)	66.8(1.7)	18.0(1.1)

**Table 2:** Composition of the different samples determined by EDX analysis

	Ni	Mn	Sn
Ni_50_Mn_45_Sn_5_	50.3	44.7	5.0
Ni–Mn	49.6	50.4	
Ni_2_MnSn	49.2	24.2	26.6

**Fig. 2:**
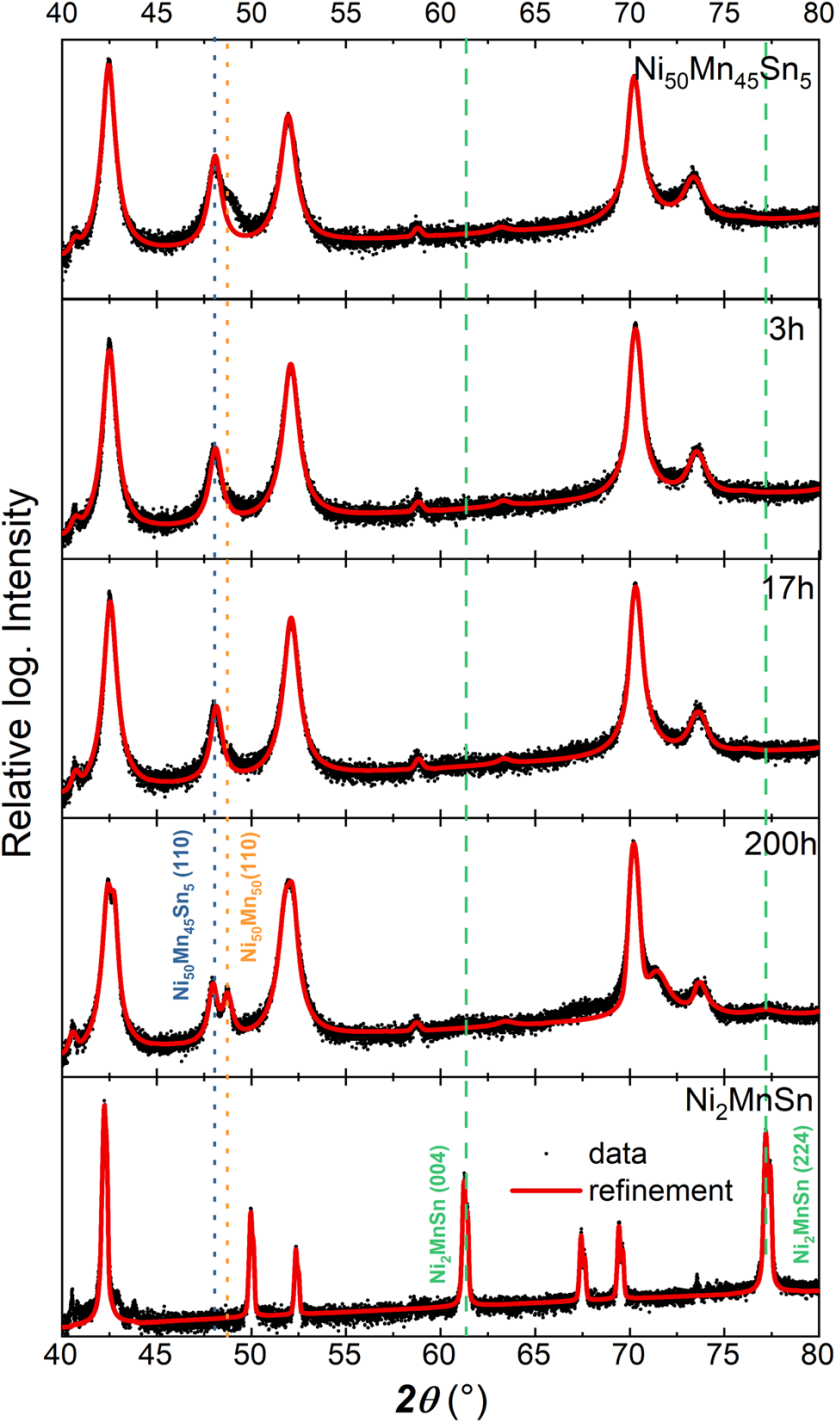
Comparison of XRD diffractograms (black circles) for the different annealed states and the corresponding refinements (red line). Certain bragg peaks that can be used as a finger print of the respective phase have been labelled.

X-ray diffraction probes the long-range ordering of the lattice structure. Fig. 2 depicts the X-ray diffractograms for the decomposed states after 3, 17, and 200 h for annealing temperature *T*_A_ of 650 K. In contrast to the spectroscopy, XRD measurements have been performed on bulk ingots, while after annealing the surface has been polished. Due to the present texture of the sample and the relative small grain size of the Ni_2_MnSn-precipitates,^20,21^ the (110) and (200) superlattice peaks of the L2_1_ structure possess a small intensity. These diffractograms indicate that Ni_50_Mn_45_Sn_5_ crystallizes in its initial L1_0_-phase, similar to Ni–Mn with a small deviation of the lattice constant, while the stoichiometric compound Ni_2_MnSn possess a L2_1_ ordering. Only after annealing the sample for 200 h, the decomposition becomes visible in the XRD-pattern as a splitting of the (110) L1_0_-peaks. Additional detailed analysis shows that the (004) and (224) peaks of the L2_1_ full Heusler Ni_2_MnSn alloy are barely visible after annealing for 200 h. The small contribution of the L2_1_-phase in the XRD pattern can be explained by the relatively small precipitate size^20,21^ (below 10 nm) and the low phase fraction of the formed full-Heusler precipitates. For the investigated post-annealing conditions, the XRD patterns indicate that the long-range ordering of the sample has barely changed, while Mössbauer spectroscopy reveals drastic variations of the Sn nearest neighbour surrounding. These variations of the local surrounding are reflected in the nuclear hyperfine levels.

## 3 Summary

Within this work, we investigated the formation of Ni_2_MnSn-precipitates in off-stoichiometric Ni_50_Mn_45_Sn_5_*via* X-ray diffraction and Mössbauer spectroscopy. Here, the transferred hyperfine field (or the induced magnetic moment) of ^119^Sn is an interesting property for investigating and tracking the precipitation process. While X-ray diffraction reveals long-range ordering, ^119^Sn-Mössbauer spectroscopy probes nearest–neighbour interactions and is, therefore, especially sensitive to changes in the local surrounding. Due to these differences in the probed length scale, we can explain the different occurring dynamics. Employing extended X-ray absorption fine structure (EXAFS) spectroscopy, this concept can be adapted to different material systems. For example, one can probe the diffusion at grain boundaries^37^ in Nd_2_Fe_14_B or Sm-Co,^38^ or one can use scanning transmission X-ray microscopy^39^ and find a connection between the local structure and high coercivity occurring in high-performance permanent magnets.

## 4 Experimental details

All samples were prepared by arc melting of pure elements (Ni: 99.98%, Mn: 99.99%, Sn: 99.999%). Afterwards, the obtained material was encapsulated in a quartz tube under argon atmosphere and homogenized for five days at 1073 K, followed by quenching in room temperature water and polished. Energy-dispersive X-ray spectroscopy inside a scanning electron microscope verifies the composition of the prepared alloys (see Table 2). X-Ray diffraction measurements were performed on a polycrystalline bulk ingot using a Phillips PANalytical X'Pert PRO with non-monochromatized X-rays (Cu X-ray source) in a Bragg–Brentano geometry, and the obtained diffraction patterns were analyzed using JANA2006.^40^ For the investigation of the decomposition, Ni_50_Mn_45_Sn_5_ was annealed a temperature of 650 K for different times under high vacuum conditions (*p* ≈ 5 × 10^−5^ mbar) to avoid oxidation of the sample. During the annealing process, no magnetic field has been applied. Room temperature ^119^Sn-Mössbauer spectroscopy measurements were performed in transmission geometry under zero-field conditions with conventional electronics. The velocity of a Ca^119^SnO_3_ source was changed within the constant-acceleration mode and calibrated with a laser interferometer. The experimental spectra have been evaluated by a least-squares fitting routine using the Pi program package.^41^

## Conflicts of interest

There are no conflicts to declare.

## Supplementary Material
